# Supplementary material for: Complexome profiling of the Chlamydomonas psb28 mutant reveals TEF5 as an early PSII assembly factor
Source: Plant Cell. 2025 Mar 17;37(6):koaf055. doi: 10.1093/plcell/koaf055 (PMC12164590; doi:10.1093/plcell/koaf055)
Supplement: koaf055_Supplementary_Data [file koaf055_supplementary_data.zip › psb28_interactive.zip!/plots/intensity/qualityPlots/peptides/PGH1_Cre12.g513200.t1.2.html]

### Cre12.g513200.t1.2

Trivial name: PGH1  
SeqCoverage [median]: 14.90  
Mass: 51.60 kDa  
  
 

| Sequence | MissedCleavage | Mass [Da] | wtIntensity | muIntensity | Score |
| --- | --- | --- | --- | --- | --- |
| AAVPSGASTGVHEAVELR | 0 | 1749.90 | 759.75 | 0.00 | 64.52 |
| ALGQLTPPEIVK | 0 | 1264.74 | 712.92 | 1968.57 | 79.97 |
| AVENINAIIAPALK | 0 | 1435.84 | 623.09 | 0.00 | 101.66 |
| GNPTVEADVYTR | 0 | 1320.63 | 0.00 | 2279.98 | 97.16 |
| VNQIGTITESIEAVR | 0 | 1628.87 | 638.50 | 2725.01 | 96.82 |
|  |  |  | 2734.3 | 6973.6 |  |
